# Should a booster dose be administered in children after mass immunization for hepatitis B?

**Published:** 2011-06-01

**Authors:** Selma Tosun, Serol Deveci, Yunus Kaplan, Erhun Kasirga

**Affiliations:** 1 Manisa State Hospital, Department of Infectious Diseases and Clinical Microbiology, Manisa, Turkey; 2 Celal Bayar University, High School of Health, Manisa, Turkey; 3 Celal Bayar University, Medical Faculty, Department of Paediatrics, Division of Gastroenterology, Manisa, Turkey

**Keywords:** Hepatitis B vaccine, Immunity, Immunization, Booster

## Abstract

**Background:**

Hepatitis B virus infection is a global health problem and vaccine-preventable disease, but the duration of the effects of HBV vaccination in infants is unknown.

**Objectives:**

The aim of this trial, which comprised children who had received 3 doses as part of the universal HBV immunization program and no additional doses, was to investigate anti-HBs titers and HBsAg status after 9 years.

**Patients and Methods:**

We performed a descriptive, cross-sectional field research study. The study sample, based on sociodemographics and minimum seroprevalence, was analyzed based on 10.00% ± 1.50 (95% confidence interval) (1150 individuals); trial was realized in a total of 1279 children (623 females and 656 males). Anti-HBs titers were measured by micro-EIA (Dia Sorin-Italy); titers < 10 IU/mL were negative, 10-49 IU/mL were low-positive, and > 50 IU/mL were high-positive. For anti-HBs titers below protective levels, HBsAg was measured by micro-EIA.

**Results:**

In approximately half of the children (48.5% in those living in semiurban areas and 42.3% in urban areas), antibody titers were below protective levels.

**Conclusions:**

Mass HBV vaccination, which was implemented in Turkey in 1998, significantly decreases HBsAg positivity in childhood. Nevertheless, it might be necessary to administer a booster dose after 8-9 years in children, especially those in low socioeconomic areas or in whom irregular/insufficient immunization is suspected.

## 1. Background

A number of vaccines have been used since 1982 to prevent hepatitis B virus (HBV) infection. Universal HBV immunization is regarded as an efficient and easily implemented method and has been recommended by the World Health Organization since 1997 to be given after birth in all countries. HBV immunization, which is currently performed in many countries, was initiated in 1998 in Turkey. A total of 3 doses of the HBV vaccine is recommended in individuals who do not have any immune system impairments; no booster dose was deemed to be required. However, some trials that have concluded that antibody levels in infants who are immunized at birth decrease after 6-7 years and that a booster dose is required [1][2].

## 2. Objectives

Our aim was to measure anti-HBs titers and HBsAg status after 8-9 years in children who received 3 doses in the mass HBV immunization program, which began in 1998 in Turkey and no additional boosters.

## 3. Patients and Methods

### 3.1. Design, sample, and data collection

Our trial was a descriptive and cross-sectional field study. The study population was 4577 third-grade primary schools students in Manisa City Center, based on 2009 data from the Manisa City Directorate of National Education. Study sample of the trial- according to any sociodemographic characteristic, estimation of minimum level of the minimum seroprevalence category-was calculated on the basis of 10.00% ± 1.50% (95% Confidence Interval) (1150 individuals); trial was realized in a total of 1279 children, 623 females and 656 males. The subjects were selected randomly by stratified set sampling method. Thirty-nine primary schools in the city center was weighted based on their status as a public-private and urban/semi-urban school. Third grade students in the trial received an HBV vaccination at birth and no booster doses. A survey was completed by the parents, who gave informed consent for us to obtain blood samples from their children.

### 3.2. Variables

The dependent variables of the trial were HBV indicators, and the independent variables were date of birth, gender, socioeconomic level, health insurance, place of birth, maternal place of birth, and residence.

### 3.3. Laboratory analysis

Per the Ethical Committee and Ministery of Health Basic Health Services General Directorate and with consent from the parents, schools were visited in February and March 2009. A 5-6-cc blood sample was obtained from every student, and anti-HBs titers were measured by micro-EIA (Dia Sorin-Italy). Anti-HBs titers < 10 IU/mL were regarded as negative, 10-49 IU/mL were low-positive, and > 50 IU/mL were high-positive. For cases with anti-HBs titers below protective levels, HBsAg was measured by micro-EIA (Dia Sorin-Italy).

### 3.4. Data analysis

Descriptive statistics, Pearson chi square, t-test, and one-sided variance analysis (ANOVA) were used to analyze the data using SPSS 10.0.

## 4. Results

### 4.1. Sample characteristics

**Figure 1:**
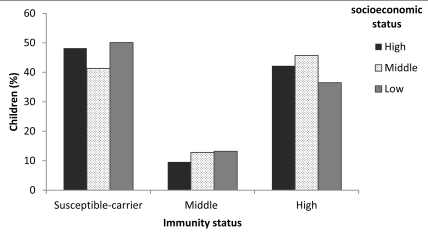
Immunity Status by Socioeconomic Level in Children (%)

The number of children in the trial was 1279, comprising 51.3% (656) boys and 48.7% (623) girls. The percentages of children from upper, middle, and lower socioeconomic levels were 8.9% (114), 47.7% (610), and 43.3% (555), respectively. The immunity of children by socioeconomic characteristics is shown in Table 1, and their susceptibility to risk of hepatitis B is listed in Table 2. The immunity status of the participants by socioeconomic level is shown in (Figure 1).

**Table 1:** Immunization among children according by sociodemographics^a^

**Demographic characteristic **	**HBsAg **(+) No. (%)	**Anti-HBs IU/mL **No.(%)			**Arithmetic a**Mean ± SD	**Statistics**	**Total**
		**< 10 IU/mL**	**10-49 IU/mL**	**> 50 IU/mL**
**Socioeconomic level**							
**High **	_	55 (48.2)	11 (9.6)	48 (42.2)	1.33 ± 0.96	p = 0.006	114
**Middle**	1 (0.2)	252 (41.3)	78 (12.8)	279 (45.7)	1.43 ± 0.93	f = 5.12	610
**Low**	1 (0.2)	278 (50.1)	73 (13.2)	203 (36.5)	1.26 ± 0.94	_	555
**Health insurance **							
**Yes**	1 (0.1)	392 (43.7)	110 (12.3)	393 (43.9)	1.39 ± 0.95	p = 0.020	896
**No-Green Card**	1 (0.3)	187 (50.7)	48 (13.0)	133 (36.0)	1.25 ± 0.92	t = 2.33	369
**Residence **							
**Urban**	1 (0.2)	208 (42.1)	69 (14.0)	216 (43.7)	1.43 ± 0.91	P = 0.012	494
**Semi urban**	1 (0.1)	327 (48.2)	79 (11.6)	272 (40.1)	1.29 ± 0.96	t = 2.51	679
**Place of birth **							
**Manisa-Izmir**	1 (0.1)	472 (44.4)	137 (12.9)	452 (42.6)	1.37 ± 0.93	p = 0.012	1062
**Other**	1 (0.5)	108 (53.8)	22 (10.9)	70 (34.8)	1.19 ± 0.96	t = 2.52	201
**Place of birth of mother **							
**Manisa-Izmir**	_	207 (40.3)	72 (14.0)	235 (45.7)	1.45 ± 0.91	p = 0.002	514
**Other**	2 (0.3)	363 (49.3)	86 (11.7)	285 (38.7)	1.28 ± 0.96	t = 3.07	736
**Total**	2 (0.2%)	585 (45.7%)	162 (12.7%)	530 (41.4%)	1.35 ± 0.94	_	1279

^a^ Analysis was performed by log10-transforming anti-HBs titers

**Table 2:** Hepatitis B susceptibility by sociodemographics

***Variable***	** Immunity status in children**		***OR *****a** (95% CI)	***P value *****b**
	**Carrier-susceptible-low-levelimmunity** No. (%)	**Middle-high level immunity **No. (%)		
**Socioeconomic level**				0.005
**Middle-high (n = 724)**	308 (42.5)	416 (57.5)	1.37	
**Low (n = 555)**	280 (50.5)	275 (49.5)	(1.10-1.71)	
**Health insurance**				0.017
**Yes (n = 896)**	393 (43.9)	503 (56.1)	1.34	
**No–Green Card(n = 369)**	189 (51.2)	180 (48.8)	(1.05-1.71)	
**Residence**				0.037
**Urban (n = 494)**	209 (42.3)	285 (57.7)	1.28	
**Semi-urban.(n = 679)**	329 (48.5)	350 (51.5)	(1.01-1.61)	
**Place of delivery**				0.025
**Health institute (n = 1059 )**	473 (44.7)	586 (55.3)	1.40	
**Home-other (n = 207)**	110 (53.1)	97 (46.9)	(1.04-1.89)	
**Place of birth**				0.012
**Manisa-İzmir (n = 1062)**	474 (44.6)	588 (55.4)	1.47	
**Other (n = 201)**	109 (54.2)	92 (45.8)	(1.08-1.98)	
**Place of birth of mother **				0.001
**Manisa-İzmir (n = 514)**	207 (40.3)	307 (59.7)	1.46	
**Other (n = 736)**	366 (49.7)	370 (50.3)	(1.16-1.84)	

^a^ Odds ratio

^b^ Chi-square

### 4.2. Hepatitis B immunity status by socioeconomic level

In approximately half of the children who were vaccinated at birth in mass HBV immunization program, antibody titers were below protective levels-48.2% in the upper socioeconomic level, 41.3% in the middle level, and 50.1% in the lower level. These differences were significant, and the best antibody levels were observed in children from the middle socioeconomic level (p = 0.009).

### 4.3. Relationships between study variables

No relation was noted between family income or gender and protective antibody level. A significant difference was observed between children who were born in health institutes (55.3%) and elsewhere (46.9%) (p = 0.025 OR:1.40 95% CI:1.04-1.89). Immunity levels levels differened significantly between the low and middle-upper socioeconomic levels (p = 0.005 OR:1.37 95% CI:1.10-1.71). No correlation was observed between having a hepatitis patient/carrier at home, hepatitis history, or hepatitis family history and immunity levels.

Antibody titers were below protective levels in 43.7% of those with health insurance and in 50.7% in those without it or holders of green card insurance (p = 0.017 OR:1.05 95% CI:1.05-1.71) Anti-HBs titers were below protective levels in 48.5% of people who were living in semiurban areas and 42.3% in those in urban areas.

(p = 0.037 OR:1.28 95% CI:1.01-1.61). The percentage of children who were born in Manisa or Izmir with anti-HBs titers < 10 IU/mL was 44.6% versus 54.2% for those born elsewhere (p = 0.012 OR:1.47 95% CI:1.08-1.98). Similarly, 40.3% of children whose mothers were born in Manisa or Izmir had anti-HBs < 10 IU/mL compared with 49.7% of children whose mothers were born elsewhere (p = 0.001 OR:1.46 95% CI:1.16-1.84).

## 5. Discussion

Mass hepatitis B immunization has been successful in a number of trials [3][4][5][6], This program, which is widespread and being implemented in many countries, was adopted in Turkey for the routine immunization of newborns and risk groups in August 1998. Certain revisions in the immunization schedule were made, as indicated in circulars on this issue; the latest revisions recommeded the first dose to be administered at birth, the second dose at 1 month, and last dose 4 months after the second dose. In terms of protection against hepatitis B infection, when antiHBs titers exceed 10 mIU/mL, even if it decreases later, protection against hepatitis B infection continues; in babies who are vaccinated at birth, booster doses are not required for at least 5 years, and there are sufficient immune memory cells that effect good responses to boosters [6][7][8][9][10].

The World Health Organization states that boosters are not required, following properly administered hepatitis B vaccinations. Recommendations of the European Consensus Group on Hepatitis B Immunity also opine that no booster is required in individuals with complete immunization and in those who do not have any immune-related problems [11]. However, some trials have indicated that following vaccination at birth, the anti-HBs response decreases over time and that a booster dose is required. In a study in which blood samples were drawn from children at age 5 years who were given a yeast-based vaccine at birth, only 26 (12.5%) of 208 children had a seroprotective anti-HBs response; in the 7-year evaluations, no seroprotective response was observed in any of 36 children who were tested. When 1 booster was administered to these children, a protective response with a ratio of 90% was seen in both groups. The trial authors concluded that in the majority of children who are vaccinated at birth, anti-HBs titers decline but that immunological memory exists, effecting a response very good to booster doses. They emphasized that large-scale trials are needed to determine whether booster doses are required in children who are vaccinated at birth and the timing of such boosters [12].

Similarly, in a trial in Iran, where a national HBV immunization program was initiated in 1993, of the 3758 children who were vaccinated in this program, 19.3% had anti-HBs levels < 10 IU/mL, 51.6% was between 10-100 IU/mL, and 29.2% was > 100 IU/mL [13]. In a trial in Manisa in 2008, HBV seroprevalence before and after immunization was examined among primary school students, and hepatitis B vaccine was administered to children before or after blood samples were drawn in the immunization program of the Ministery of Health. The study group observed that 70.7% of children who received the booster dose were immune (No.:571) versus 34.3% (No.:239) of children who did not (x² = 200.119, p = 0.000). As expected, anti-HBs titers in children who received reminder doses were also significantly higher (p = 0.000, t = 18.58) versus those who did not [14].

We observed that in children who were vaccinated at birth in 2000 and received no boosters, 48.2%, 41.3%, and 50.1% of those in the high, middle, and low socioeconomic levels were negative (< 10 IU/mL) for anti-HBs responses, respectively; 9.6%, 12.8%, and 13.2% had low anti-HBs (10-49 IU/mL) responses, respectively, and 42.2%, 45.7%, and 36.5% had high titers, indicating that antiHBs titers decrease 8-9 years after mass immunization. Two hundred ninety of 585 children with anti-HBs titers < 10 IU/mL were contacted again and given and 1 dose of HBV. One month later, all but 5 had high antiHBs titers; in these 5 children, 1 experienced immunosupression, 1 was mentally retarded, and the remaining 3 had not been immunized since birth. These results indicate that primary immunization in a universal HBV vaccination program is favorable but that anti-HBs titers decrease to undetectable levels over time. A meta-analysis from Iran of 34 studies that comprised 9356 individuals concluded that additional studies are needed to assess vaccine efficacy for longer periods, as are booster doses in various subgroups [15].

The lack of protective anti-HBs production, despite HBV vaccination, can result, possibly from noncompliance with cold chain procedures; administration of vaccine to an inappropriate area of the body; incorrect vaccination procedure; loss of efficacy of the vaccine, which is especially susceptible during freezing; failure to administer the vaccine at birth; delay in vaccination; and a having an HbsAg-positive mother. In addition, the initial administration of HBV vaccine in Turkey varied with regard to start time, which might have affected the long-term antibody response. Thus, a booster dose should be given 8-9 years after universal immunization if a child is born in an endemic area, if the mother or any family member shows HBsAg positivity, if the vaccination history of the child is unknown, if there is any uncertainty with regard to the storage/administration of the vaccine, or if the child lives in a low socioeconomic area. In addition, HBsAg should be measured in all pregnant women, especially those from low socioeconomic areas, and be recorded in their follow-up information, so that vaccine + HBIG can be administered to babies who are born to carriers and so that mothers who deliver at home can be identified as soon as possible to enroll the babies in the immunization program.

In conclusion, mass HBV immunization, which was begun in 1998 in Turkey, has significantly decreased HBsAg positivity, especially during childhood. However, for children who are suspected of having irregular/insufficient immunization or those who live in low socioeconomic areas, a booster dose might be necessary 8-9 years after primary immunization.
